# The role of the Cx43/Cx45 gap junction voltage gating on wave propagation and arrhythmogenic activity in cardiac tissue

**DOI:** 10.1038/s41598-023-41796-w

**Published:** 2023-09-08

**Authors:** Kestutis Maciunas, Mindaugas Snipas, Tadas Kraujalis, Lina Kraujalienė, Alexander V. Panfilov

**Affiliations:** 1 Institute of Cardiology, Lithuanian University of Health Sciences, Kaunas, Lithuania; 2 Department of Mathematical Modelling, Kaunas University of Technology, Kaunas, Lithuania; 3 Department of Applied Informatics, Kaunas University of Technology, Kaunas, Lithuania; 4 Department of Physics and Astronomy, Ghent University, Ghent, Belgium; 5 Department of Cardiology, Leiden University Medical Center, Leiden, The Netherlands

**Keywords:** Computational biophysics, Computational models, Computer modelling, Numerical simulations

## Abstract

Gap junctions (GJs) formed of connexin (Cx) protein are the main conduits of electrical signals in the heart. Studies indicate that the transitional zone of the atrioventricular (AV) node contains heterotypic Cx43/Cx45 GJ channels which are highly sensitive to transjunctional voltage (V_j_). To investigate the putative role of V_j_ gating of Cx43/Cx45 channels, we performed electrophysiological recordings in cell cultures and developed a novel mathematical/computational model which, for the first time, combines GJ channel V_j_ gating with a model of membrane excitability to simulate a spread of electrical pulses in 2D. Our simulation and electrophysiological data show that V_j_ transients during the spread of cardiac excitation can significantly affect the junctional conductance (g_j_) of Cx43/Cx45 GJs in a direction- and frequency-dependent manner. Subsequent simulation data indicate that such pulse-rate-dependent regulation of g_j_ may have a physiological role in delaying impulse propagation through the AV node. We have also considered the putative role of the Cx43/Cx45 channel gating during pathological impulse propagation. Our simulation data show that V_j_ gating-induced changes in g_j_ can cause the drift and subsequent termination of spiral waves of excitation. As a result, the development of fibrillation-like processes was significantly reduced in 2D clusters, which contained V_j_-sensitive Cx43/Cx45 channels.

## Introduction

Gap junction (GJ) channels formed of connexin (Cx) protein mediate the propagation of action potentials (APs) between cells in cardiac tissue. In the human cardiac conduction system, GJ channels are formed from three main Cx isoforms: Cx43, Cx40 and Cx45 (see rev. in^1^). Among them, Cx43 is the most prominently expressed and Cx45 is the least. The expression patterns of these Cxs are also location-dependent. For example, the atrioventricular (AV) node preferentially expresses Cx45, while in the atrium and the ventricles, Cx43 is much more prominently expressed^2,3^. Moreover, some studies have shown sparsely distributed regions of nodal cells in which Cx43 and Cx45 colocalize^4–6^, which raises an interesting possibility that some cells may be connected through heterotypic Cx43/Cx45 channels. Electrophysiological studies have confirmed that such channels, formed of both rodent and human Cxs, would be functional^7,8^. Thus, in this study, we investigated the putative role of heterotypic Cx43/Cx45 channels in the physiological and pathological spread of cardiac excitation.

Experimental studies have established that GJ channels are modulated by transjunctional voltage (V_j_): that is, the difference of membrane voltages between apposing cells^9^. V_j_ sensitivity of GJ channels is Cx-dependent. For example, homotypic channels formed by Cx45 are much more V_j_-sensitive than those formed by Cx43. However, when these Cxs are arranged in heterotypic configuration, the resulting Cx43/Cx45 channels exhibit an asymmetric V_j_ sensitivity profile. More precisely, junctional conductance (g_j_) of Cx43/Cx45 channels decreases sharply when the Cx43 side is exposed to positive V_j_s (as sensed by the Cx43 side: i.e., the membrane potential on the cell expressing Cx43 is relatively positive), and increases moderately in response to negative V_j_s^7^. This can be explained by the contingent gating model of GJ channels, in which gating transitions depend on the V_j_ distribution across apposing hemichannels^10^, as well as on Cx-dependent sensitivity to either positive or negative V_j_s^11^. The expression patterns of cardiac Cxs indicate that during normal orthodromic excitation, heterotypic Cx43/Cx45 GJs would be exposed to V_j_s positive on the Cx43 side^8^. Thus, the g_j_ of heterotypic Cx43/Cx45 channels could be dynamically modulated by V_j_ transients that develop during impulse propagation.

Thus far, the V_j_ gating of GJ channels has rarely been accounted for in modelling studies. To our knowledge, the few studies which have addressed this topic have only considered homotypic Cx43/Cx43 channels and 1D tissues of cells^12,13^. In this study, to simulate the behaviour of heterotypic Cx43/Cx45 GJs, we applied our recently developed four-state model (4SM) of GJ channel V_j_ gating, which can adequately describe the kinetics of g_j_ of both homotypic and heterotypic GJs^14^. First, we performed electrophysiological experiments in heterogenous cell pairs consisting of Novikoff cells expressing Cx43 and HeLa cells expressing Cx45. When docked together in a cell culture medium, these cells formed heterotypic Cx43/Cx45 channels. We fitted the 4SM to the recorded transjunctional currents and obtained a set of model parameters which could adequately describe the observed kinetics of the g_j_ of the Cx43/Cx45 channels in response to the applied V_j_ gradients. The obtained set of 4SM parameters was then used in simulations of impulse propagation in a 2D tissue of discrete cells.

Typically, studies that model the spread of electrical excitation in cardiac tissue consider a continuum approach to make computation tractable^15^. However, such an approach makes it more difficult to account for the influence of GJs^16^, let alone their dynamic gating. In this study, we developed a 2D model of cardiac tissue which was composed of discrete cells interconnected hexagonally by GJ channels. Cardiac excitability was described by the Fenton–Karma model^17^, and g_j_ of Cx43/Cx45 was described using the 4SM. Other types of junctions were assumed to exhibit either a constant conductance (i.e., to mimic the properties of less V_j_-sensitive homotypic Cx43 GJ channels) or non-conductivity (i.e., to mimic the properties of collagenous strands, vessels or fibroblasts). To increase the speed of the computation, we applied parallelization on a graphics processing unit (GPU) and optimized the data structures.

Our simulation experiments revealed that a series of V_j_ transients, which developed across the Cx43/Cx45 channels during the orthodromic spread of excitation, could significantly reduce g_j_. This g_j_ decrease was pulse-rate-dependent and was more prominently expressed at higher frequencies of excitation. This data was supported by our and other authors’ electrophysiological recordings^8^. We hypothesize that such a pulse-rate-dependent g_j_ decrease may have a physiological role. For example, it could increase the delay of impulse propagation through the AV node, which is necessary for maintaining normal blood flow into the ventricles. The viability of such a mechanism was demonstrated by our simulations in a 2D cluster of cells. They showed that the V_j_ gating-induced g_j_ decrease of a single heterotypic Cx43/Cx45 GJ can delay the impulse propagation by up to ~ 5 ms, and this delay can add up with each heterotypic junction.

In addition, we evaluated the potential role of the Cx43/Cx45 channel V_j_ gating during pathological impulse propagation. Due to the presence of slow and high-conductance pathways, the AV node is susceptible to one of the most common re-entrant arrhythmias, called atrioventricular node re-entry tachycardia^18,19^. Thus, we evaluated if the V_j_ gating could affect the dynamics of the spiral waves of excitation which underlie re-entrant arrhythmias^20^. We presumed that the Cx43/Cx45 GJs located on the trajectory of the spiral waves would be exposed to V_j_ transients of very high frequencies; therefore, it could exhibit significant g_j_ changes. Our simulation data show that such V_j_ gating-induced inhomogeneities of g_j_ can cause the drift of a spiral wave. Such a drift is thought to have an antiarrhythmic effect due to the termination of the spiral waves after their collision with the anatomical border^21^. We also evaluated the effect of V_j_-induced gating on the development of complex fibrillation-like processes, which are characterized by the spread of multiple unstable spiral waves. Our simulation data indicate that heterogenous 2D clusters, which contained V_j_-sensitive heterotopic Cx43/Cx45 GJs, have exhibited a statistically significant reduction in arrhythmogenic activity, as compared to identical clusters connected only through non-gated GJs.

Overall, we conclude that the g_j_ of heterotypic Cx43/Cx45 GJ channels can be affected by V_j_ transients, which would develop during cardiac impulse propagation. The pulse-rate-dependent nature of such V_j_ gating-induced g_j_ modulation suggests possible physiological roles of this mechanism, including an increased delay of impulse propagation through the AV node or reduction of arrhythmogenic activity through the termination of re-entrant activity.

## Results

### The evaluation of the V_j_ gating properties of the heterotypic Cx43/Cx45 GJ channels

**Figure 1:**
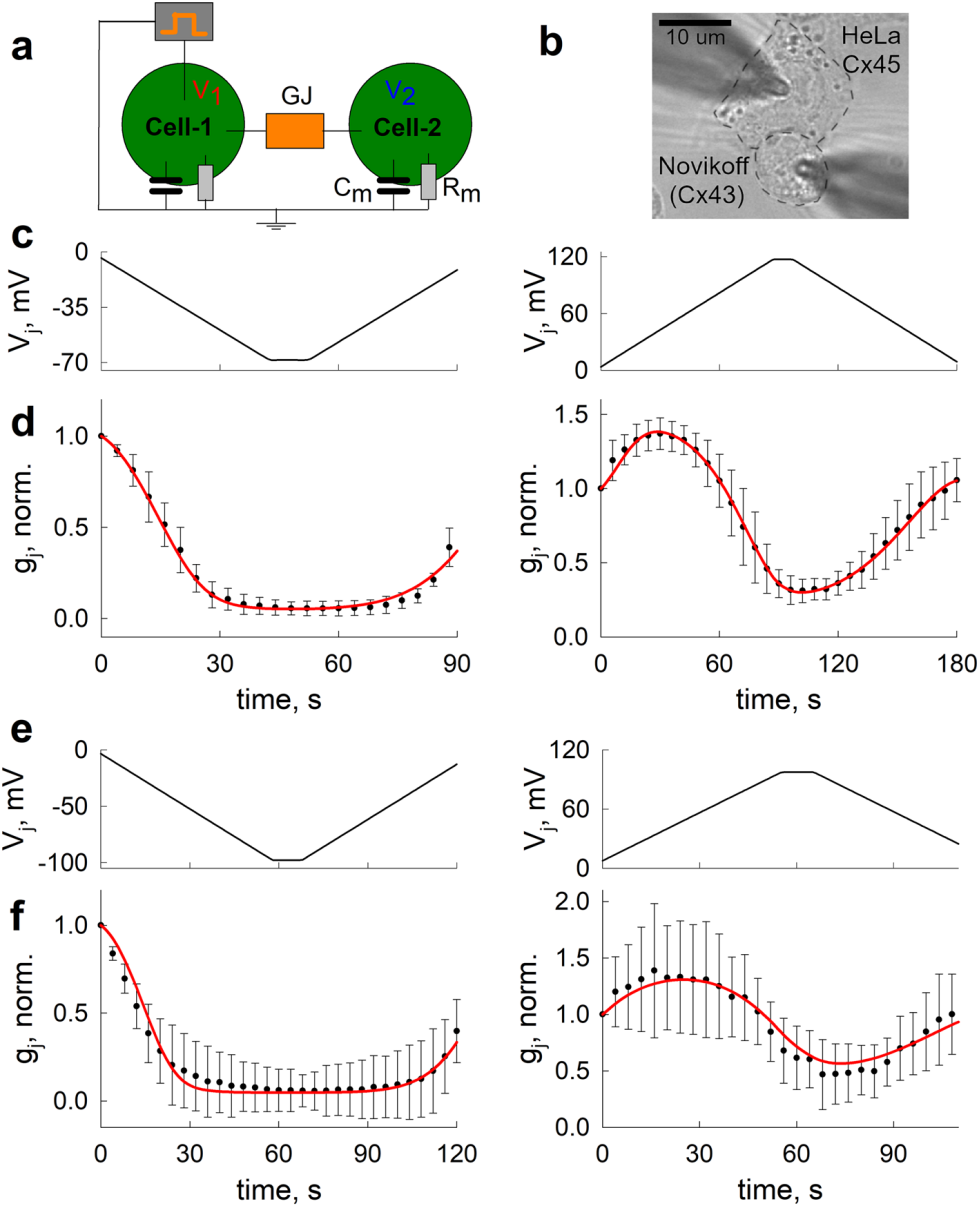
Model fitting of the 4SM to electrophysiological data obtained from heterotypic Cx43/Cx45 channels. (**a**) The schematics of the dual whole-cell patch clamp experiment. Apposing cells are connected through a V_j_-sensitive GJ. (**b**) Phase contrast image of the smaller, Cx43-expressing Novikoff, and the larger, Cx45-expressing HeLa cell (cell membranes are highlighted by the dashed lines) during the dual whole-cell patch clamp experiment. Patched pipettes are shown in dark grey. (**c**,**e**) are the ramp protocols used in electrophysiological experiments for the evaluation of the V_j_ gating parameters of the Cx43/Cx45 channels. (**d**,**f**) are the respective averaged g_j_ time courses (whisker plots) obtained from at least five different recordings; the error bars denote standard deviations. The solid red lines show the theoretical g_j_ and the time courses obtained using the 4SM. The model parameters were obtained by fitting the 4SM to the data presented in (**d**). The same set of parameters was used in generating the theoretical g_j_ time courses in (**f**), which provide additional validation of the model.

To evaluate the V_j_ gating properties of the heterotypic Cx43/Cx45 GJ channels, we performed electrophysiological experiments in cell cultures (see Fig. 1a,b). We recorded the kinetics of the g_j_ (Fig. 1d,f) of the Cx43/Cx45 GJ channels in response to V_j_ ramps of different lengths and steepness (Fig. 1c,e). The g_j_ substantially decreased with an increase of positive V_j_ and the changes in g_j_ depended on the speed at which the V_j_ varied. Data averaged from at least five different recordings (whisker plots in Fig. 1d) were used for the estimation of the 4SM parameters, which would describe the V_j_ gating properties of the Cx43/Cx45 channels. Model fitting was performed using global optimization methods, mainly dual annealing and differential evolution algorithms. The solid red lines in Fig. 1d show the g_j_ time courses simulated using the obtained set of 4SM parameters. The obtained parameters were additionally validated by electrophysiological recordings (Fig. 1f), for which different V_j_ ramps were used (Fig. 1e). These datasets were not used in the model fitting. Because the obtained set of 4SM parameters showed good fits with both the model training (Fig. 1d) and validation (Fig. 1f) datasets, we suggest that the 4SM is capable of adequately describing the kinetics of the heterotypic Cx43/Cx45 GJs in response to the applied V_j_s of various amplitudes and time courses.

### V_j_ transients developed during the spread of cardiac excitation can cause the g_j_ decrease of Cx43/Cx45 GJs in both simulation and electrophysiological experiments

**Figure 2:**
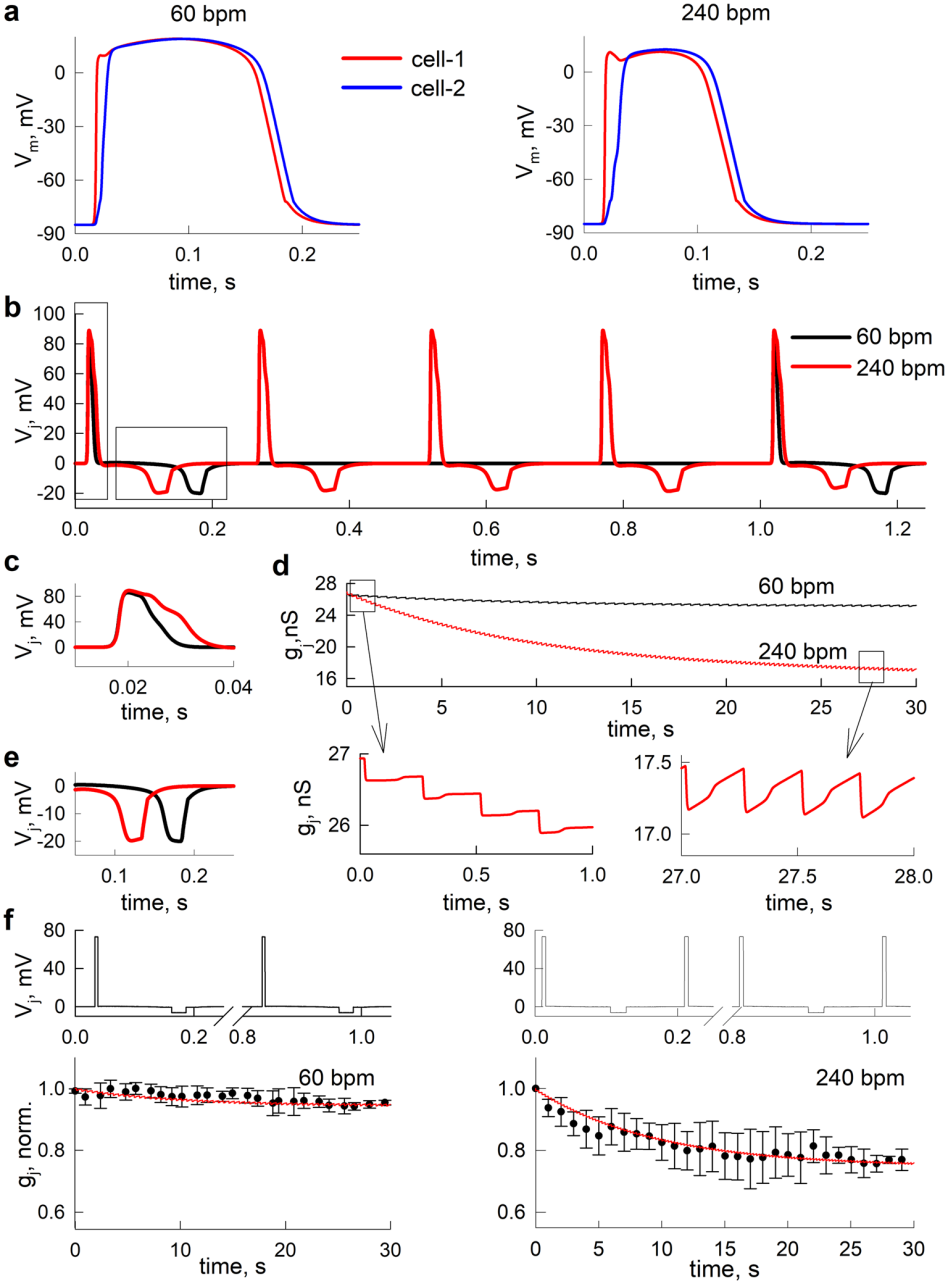
The decrease of g_j_ in response to V_j_ transients which develops during the spread of excitation in the cardiac tissue. (**a**) The APs of two adjacent cardiomyocytes in the simulated 2D cardiac tissue. (**b**) The respective transjunctional voltages (V_j_), which would be sensed by a connecting GJ. Insets (**c**,**e**) show the enhanced view of two distinct phases of a biphasic V_j_ transient. (**d**) The simulated kinetics of the g_j_ decrease of Cx43/Cx45 channels caused by the series of V_j_ transients (presented in **b**) developed during the spread of cardiac excitation. The inserts below show the cumulative decrease of g_j_ during the initial phase (left insert), and the fluctuation of g_j_ around a new baseline level after a conditional steady state was reached (right insert). (**f**) The stylized V_j_ protocols (upper panels), resembling a series of V_j_ transients developed during the spread of excitation which were used in our electrophysiological experiments. The lower panels show the recorded changes of average g_j_ (black circles), obtained in response to the stylized V_j_ protocols. The solid red lines show the respective theoretical g_j_ time course which is obtained using the 4SM. The error bars denote the standard deviations.

The obtained set of 4SM parameters, which describe the kinetic properties of the V_j_ gating of the Cx43/Cx45 channels, allowed us to evaluate the complex interdependence between the GJ channel gating and the propagation of cardiac excitation using our combined model of cardiac tissue. First, we evaluated the differences in membrane potentials between the adjacent cardiomyocytes (i.e., V_j_), which would develop during the propagation of excitation at different pulse rates. Figure 2a shows the APs of two adjacent cardiomyocytes obtained from simulations with different pulse rates: normal (60 bpm, left panel in Fig. 2a) and very high (240 bpm, right panel in Fig. 2a). Figure 2b shows the overlay of the respective V_j_ transients that developed during the spread of cardiac excitation. It can be seen that the V_j_ transients are biphasic and consist of a relatively short (~ 8–16 ms), high-amplitude positive (~ 85 mV) V_j_, which is followed by a longer (~ 30 ms), but lower-amplitude (~ -20 mV) negative V_j_. Moreover, there is a visible difference between the V_j_ transients developed during the two pulse rates. Mainly, at 240 bpm, the positive V_j_ phase is more prominently expressed and the negative V_j_ phase follows it after a shorter delay compared to the V_j_ transients developed at 60 bpm (inserts in Fig. 2c,e). Figure 2d shows an overlay of the resulting g_j_ time courses in response to these series of V_j_s. At the beginning of the simulations, when a high proportion of Cx43/Cx45 channels was still open (g_j_ was equal to a steady state value obtained at V_j_ = 0 mV), the positive phase of the V_j_ transient can cause a small g_j_ decrease, which is followed by an even smaller g_j_ recovery (Fig. 2d, left insert). These small overall decreases of g_j_ can accumulate during the repeated propagation of excitation until a conditional steady state is reached. At this stage, the proportion of closed channels during the depolarization phase is equal to the proportion of opened channels during the hyperpolarizing phase of V_j_ transients; therefore, the g_j_ decreases and the recoveries compensate each other (Fig. 2d, right insert). Most importantly, the magnitude of the overall g_j_ decrease is pulse rate-dependent. That is, at 60 bpm, the new equilibrium g_j_ was only ~ 3% lower than at the beginning of the simulation, while at 240 bpm the respective g_j_ decrease reached ~ 30%. Thus, our data show that g_j_ is not necessarily constant, as has been typically assumed in cardiac modelling studies, but can be strongly dependent on the stimulation frequency. After the increase of the stimulation frequency, g_j_ can decrease with a characteristic time of 10–15 s.

To validate the observed modelling data, we performed electrophysiological experiments using the stylized V_j_ protocols (see upper panels in Fig. 2f), which resemble the series of V_j_ transients observed in our modelling data. The lower panels of Fig. 2f show the averaged g_j_ time courses (black circles with error bars) from five experiments, recorded in the Cx43/Cx45 GJs in response to the stylized cardiac protocols. The solid red lines show the respective g_j_ time courses, which were obtained using the 4SM and the same set of parameters as in Figs. 1 and 2d. The simulated g_j_ time courses show a reasonably good correspondence to the electrophysiological data. Thus, the 4SM can adequately reproduce the kinetics of g_j_, which could occur in response to a series of short V_j_ transients developed during cardiac excitation. Overall, our data show that V_j_ transients, developed during a cardiac excitation could regulate the g_j_ of the heterotypic Cx43/Cx45 GJ channels in a pulse-dependent manner.

### The pulse-rate-dependent g_j_ decrease of Cx43/Cx45 GJs can modulate the delay of impulse propagation in the AV node

**Figure 3:**
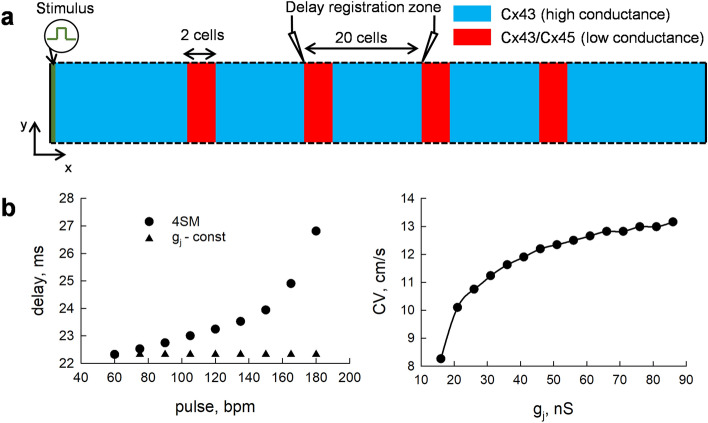
The pulse-rate-dependent decrease of g_j_ of the Cx43/Cx45 channels and its effect on the conduction velocity. (**a**) The schematics of the 2D cluster of cells, containing high (blue) and low (red) conductance zones which were used in the simulation experiments. The homotypic Cx43 channels were assumed to exhibit a constant g_j_, while the g_j_ kinetics of the Cx43/Cx45 channels were evaluated using the 4SM. (**b**) The left panel shows the dependence of the delay of impulse propagation on the pulse rate through the registration zone. The g_j_ of the heterotypic Cx43/Cx45 channels in a low-conductance zone was either constant 30 nS (black triangles) or was evaluated using the 4SM with initial g_j_ = 30 nS (black circles). The right panel shows the dependence of the conduction velocity (CV) on the g_j_ of the Cx43/Cx45 channels. The CV was measured in the registration zone (see panel a), which contained a single zone of high (300 nS) and a single zone of low (iterated from 15 to 90 nS) conductance.

We hypothesize that the observed dependence of the g_j_ of the Cx43/Cx45 channels on the pulse rate may have a physiological role, mainly in the regulation of impulse propagation through the AV node. That is, the expression patterns of cardiac Cxs indicate that the transitional cells of the AV node may contain heterotypic Cx43/Cx45 GJ channels. Moreover, these channels would be arranged in such a direction that the spread of excitation should cause a g_j_ decrease^8^. Because the delay of impulse propagation through the AV node is necessary for normal blood flow into the ventricles, it is conceivable that the pulse-rate-dependent modulation of g_j_ and, consequently, of the conduction velocity, may play a role in this process. To test this hypothesis, we performed numerical simulations in a 2 × 100 cluster of cardiomyocytes with periodic boundaries in the transverse direction. The cluster contained zones of higher conductance (300 nS) in which cardiomyocytes there connected through homotypic Cx43 GJs, and zones of lower conductance (30 nS) in which cardiomyocytes were connected through heterotypic, highly V_j_-sensitive Cx43/Cx45 GJs (Fig. 3a). The existence of higher and lower conductance zones separated by small spatial intervals is common in cardiac tissues under normal physiological conditions, and can be even more pronounced during some pathologies^22–25^.

In our simulations, we first evaluated the dependence of conduction velocity on the g_j_ of heterotypic Cx43/Cx45 channels. In addition, since our data indicate that small V_j_ transients do not cause a significant g_j_ decrease of the homotypic Cx43 channels from the baseline level of 300 nS, for simplicity, we presumed that higher conductance zones exhibit constant g_j_. The right panel in Fig. 3b shows that a ~ 30% reduction in g_j_ of heterotypic Cx43/Cx45 channels, which was observed in our simulation experiments, could result in a significant decrease in conduction velocity. Moreover, the dependence is nonlinear as such a decrease would be more robustly expressed at the lower g_j_. As was shown by our numerical experiments (Fig. 2d), the conditional steady state g_j_ of the Cx43/Cx45 channels is pulse-rate-dependent. Thus, we evaluated how the pulse rate may affect the conduction velocity of impulse propagation through the low-conductance zones which contain Cx43/Cx45 GJs. The dependence of the delay of impulse propagation on the pulse rate is shown in the left panel of Fig. 3b (black circles). It can be seen that a high (180 bpm) pulse-rate-induced g_j_ decrease of the Cx43/Cx45 channels in a single low-conductance zone can slow the impulse propagation by ~ 5 ms compared to a normal 60 bpm pulse. Note that the observed effect is solely due to V_j_ gating, as can be seen from the respective values of delays (black triangles in the left panel of Fig. 3b) obtained from the simulations in which the g_j_ of the heterotypic Cx43/Cx45 channels was kept at a constant 30 nS level. Thus, since such delays would accumulate with each Cx43/Cx45 GJ, it is conceivable that this process could play a physiological role. That is, at very high pulse rates, the V_j_ gating-induced g_j_ decrease of the heterotypic Cx43/Cx45 channels could slow down the propagation of excitation through the AV node, thus increasing the time for the flow of blood into the ventricles before the contraction.

### The V_j_ gating-induced g_j_ decrease of the Cx43/Cx45 GJs can cause the drift of spiral waves in non-homogenous cardiac tissue

**Figure 4:**
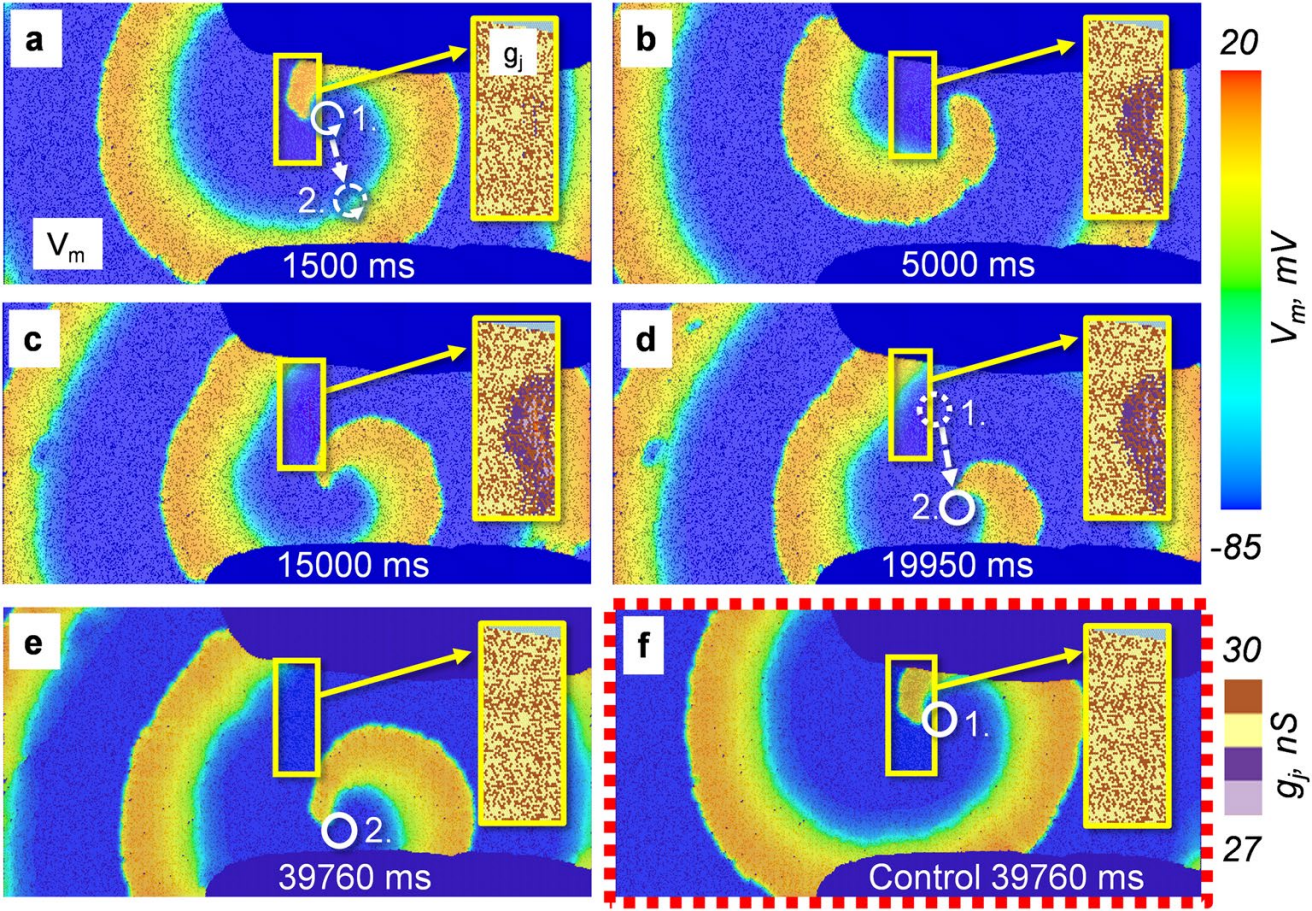
The V_j_ gating-induced drift of the spiral waves of excitation. (**a**,**b**) The initial location of the rotor (denoted by 1) in the simulated tissue of cells. A small region which contains heterotypic Cx43/Cx45 GJs is marked by a yellow rectangle. The cells outside this region were connected through either high-conductance Cx43 GJs or by non-conductive junctions. All these junctions were randomly distributed across the tissue at a ratio of 0.8:0.2. (**c**) After ~ 15,000 ms the rotor of the spiral wave started to move downwards. (**d**,**e**) The drift of the rotor stopped at ~ 19,000 ms outside the marked zone (new position denoted by 2), which contained Cx43/Cx45 GJs, where it remained stationary for the rest of the simulation. The inserts in (**a**–**e**) show the V_j_ gating-induced changes of g_j_ inside the marked zone. (**f**) No drift was observed under control conditions, with only non-gated GJs in all the clusters.

We also hypothesized that the V_j_ gating-induced changes in g_j_ of the heterotypic Cx43/Cx45 GJs could affect the spread of excitation under various pathological conditions, mainly re-entrant-type arrhythmias, to which the AV node is known to be susceptible due to the existence of two distinct conduction pathways^19^. For this purpose, we performed computational modelling experiments to evaluate the dynamics of rotating spiral waves in the tissues connected through modulatable GJ channels. Because the cells located in the domain of the spiral wave rotation should exhibit very high frequencies of excitation, we presumed that the g_j_ of the connecting heterotypic Cx43/Cx45 channels could be significantly affected. To test this hypothesis, we constructed a computational model of cardiac tissue (250 × 500 cells) which would contain the Cx43/Cx45 GJs. To mimic the non-homogeneity of real cardiac tissues, some cardiomyocytes were connected by two other types of GJs: high-conductance (~ 300 nS g_j_) homotypic Cx43 and non-conducting junctions. High-conductance and non-conducting junctions were distributed randomly across the tissue, while the Cx43/Cx45 channels were only located in the small area (marked as yellow rectangles in Fig. 4) that mimicked the transitional tissue of the AV node in our model of 2D tissue. The initial g_j_ of heterotypic Cx43/Cx45 GJs was equal to 30 nS. The ratio of high-conductance and non-conducting GJs was 0.8:0.2, with similar ratios being reported in^22,26^. In addition, in these simulation experiments, the parameters of the Fenton–Karma model were modified to adjust the restitution slopes of action potential so that the wave-front of excitation did not break and a single spiral rotor could form^27^.

In the performed simulations, we observed the drift of the generated spiral wave core out of the Cx43/Cx45 rich area. Figure 4 and the Supplementary video S1 show a representative example of this phenomenon. A more detailed analysis of spiral wave dynamics in the presented example indicates that this process was driven by the g_j_ decrease of the Cx43/Cx45 channels. That is, the cells nearest to the vortex are exposed to higher frequencies of excitation compared to the cells located at the periphery of the spiral wave. Thus, because the g_j_ decrease of modulatable GJs is frequency-dependent, the most significant changes in g_j_ are also observed near the tip of the spiral wave. Such differential changes in the g_j_ cause small variations in the velocities as the wave rotates, which can cause a small drift with each revolution of the wave. Our simulation data show that the start of the drift occurs only after some specific time (~ 15 s in the presented example) has passed (Fig. 4d), presumably when the gradients of the g_j_ reach some threshold level. Immediately after this moment, the drift increases until the spiral wave is driven outside the Cx43/Cx45-rich area. The location of the spiral wave stabilized when it was driven to the zone where the g_j_ values are constant (Fig. 4e). A similar process was consistently observed in different simulations of the 2D clusters with a similar pattern of GJ connectivity. In contrast, no drift of the spiral waves was observed in the control experiments in the 2D clusters connected only through non-gated GJs (Fig. 4f and Supplementary video S1).

### The V_j_ gating of Cx43/Cx45 reduces the probability of the formation of fibrillation-like processes in the numerical simulation of cardiac tissues

Our numerical simulations demonstrated that the V_j_ gating of Cx43/Cx45 GJs can cause the shift of the spiral wave of excitation. This process is associated with the reduction of re-entrant activity and, thus, the reduced arrhythmogenicity of the cardiac tissue. For example, the disruption of re-entry by a drift of a spiral wave tip and its subsequent collision with a non-conductive boundary has been reported in multiple studies^28–30^. We wanted to test whether the drift of the spiral waves may affect the formation of chaotic fibrillation-like processes, which exhibit a multiplication of the spiral waves. For this purpose, we performed a numerical simulation in multiple non-homogenous 2D clusters of 250 × 500 cells. To generate the histological pattern of cardiomyocytes and non-conducting junctions (fibroblasts), we used a model of virtual cardiac tissue (VCT) monolayers^31^. In addition, we included two areas of non-conducting tissue at the bottom and at the top of the cluster to mimic the central fibrous body, as well as several small zones which contained Cx43/Cx45 GJs. During the simulations, the fibrillation-like process was induced using a standard S1-S2 stimulation protocol.

**Figure 5:**
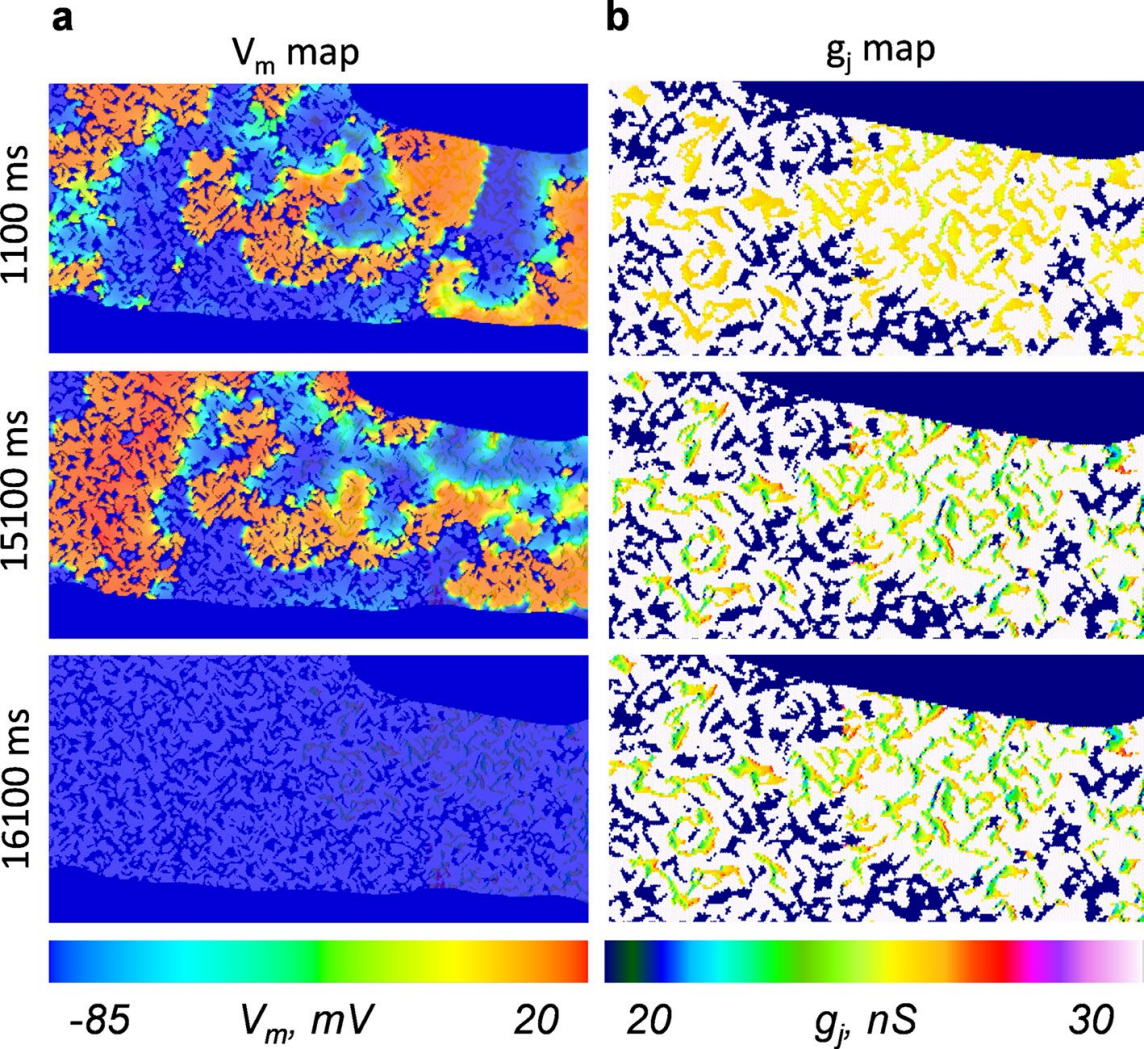
The disruption of fibrillation-like processes in the simulated 2D cluster of cells, which contain heterotypic Cx43/Cx45 GJ channels. (**a**) Snapshots of changes in the membrane potentials (V_m_) during the induced fibrillation, which terminates after ~ 16,000 ms. (**b**) The respective snapshots of changes of g_j_ in the 2D tissue containing V_j_ gated GJs, presented at slightly enhanced resolution. During the simulations under control conditions, in which all GJ channels were non-gated, termination of fibrillation was not observed.

Figure 5 and Supplementary video S2 show a representative example of a numerical experiment, in which we observed the disruption of fibrillation at ~ 16,000 ms. This disruption event seemed to correlate with the g_j_ decrease of Cx43/Cx45 GJs, which is visible in the generated g_j_ map (right middle panel in Fig. 5b). The role of the V_j_ gating-induced g_j_ decrease was verified by a control experiment (also in Supplementary video S2) in which the Cx43/Cx45 GJs were replaced by non-gated GJs. In this case, the disruption of fibrillation was not observed.

Such disruption of chaotic fibrillation-like processes may depend on multiple factors, including the geometry of the simulated tissue. Moreover, in some cases, the reduction of the arrhythmogenic activity can be more subtle and may not result in a complete interruption of the chaotic spread of excitation, at least during the limited duration of the simulation. Thus, for a more systematic evaluation of the role of Cx43/Cx45 gating in reducing the fibrillation-like activity of cardiac tissue, we performed multiple numerical experiments in differently arranged 2D tissues. More precisely, we generated 30 differently arranged 2D clusters using a VCT model with the same set of main parameters but different random seeding, and induced a fibrillation-like process in each of them under normal (i.e., the tissues contained Cx43/Cx45 GJs) and control (i.e., Cx43/Cx45 GJs were replaced with non-gated GJs of the same initial g_j_) conditions. To compare the level of the observed arrhythmogenic activity under the normal and control conditions, we estimated the modified multiscale entropy with a moving-average kernel^32,33^ of the spatiotemporal V_m_ recordings of the 2D tissue after ~ 20 s of simulation. The entropy-based analysis of mapped V_m_ data was applied in other simulation studies to describe the intensity of fibrillation and to identify the spiral wave rotors^34–36^. It can capture subtle differences in the level of disorder of the spatiotemporal V_m_ recording which would otherwise be difficult to observe by a simple visual inspection of the simulation results. The statistical analysis of performed numerical simulations showed that under the control conditions, the multiscale entropy was significantly higher than in the same tissue which contained V_j_-sensitive Cx43/Cx45 channels (the p-value of the paired t-test was lower than 0.001; the p-values of the Shapiro–Wilk test for normality were equal to 0.497 and 0.081). Thus, the obtained data indicate that the V_j_ gating-induced changes of the Cx43/Cx45 channels can significantly reduce the development of fibrillation-like processes of cardiac tissue, most likely through the disruption of re-entrant activity.

## Discussion

### Evidence of Cx43/Cx45 heterotypic GJ channels in cardiac tissue

Thus far, the most compelling evidence for the existence of heterotypic GJs in cardiac tissue is based on immunohistological and fluorescent imaging studies, which have shown the colocalization of different connexin isoforms at the same sites of cardiac tissue. For example, Cx43 and Cx45 are co-expressed at the border of the sinoatrial (SA) node and crista terminalis^37^. These results have been confirmed by other studies which have demonstrated that at the border of the SA node of a rabbit heart, atrial cells express only Cx43, most nodal cells express only Cx45, but some nodal cells express both Cx43 and Cx45^38^. (The latter observation raises an interesting possibility for the existence of heteromeric GJ channels in which hemichannels of apposing cells are composed of both C43 and Cx45^39^. However, we did not consider this possibility in this modelling study due to the lack of knowledge about the V_j_ gating properties of different heteromeric configurations.) Similar transitional structures at the border of the SA node have been observed in the cardiac tissues of mice^40^ and canine hearts^41^.

The AV node exhibits similar expression patterns of Cxs to the SA node. That is, inside the AV node there is a relative abundance of Cx45, while the surrounding tissues, such as the atrium and ventricles mostly express Cx40 and Cx43^42^. In addition, the transitional zones at the borders of the AV node in rabbit hearts show the existence of nodal cells which express both Cx43 and Cx45. Some of the observed Cx expression patterns could be consistent with the existence of heterotypic Cx40/Cx45 GJ channels, which are functional when formed from rodent Cxs, and exhibit a similar asymmetric V_j_ gating profile as heterotypic Cx43/Cx45 GJs^43^. However, somewhat surprisingly, human Cx40 and Cx45, which exhibit slightly different amino acid sequences than their respective rodent Cxs, do not form functional heterotypic GJ channels^8^.

In cell cultures, comprehensive evidence of coupling through heterotypic GJ channels can be provided not just by immunolabeling but by combined imaging and electrophysiological data, which show asymmetric changes in g_j_^7,43,44^. To our knowledge, such asymmetric gating properties have not yet been demonstrated in real cardiac tissues due to the difficulty of directly measuring the g_j_ between apposing cells, which are connected into a functional syncytium. In this study, we present indirect evidence for the existence of such heterotypic GJ junctions based on their putative functional role. Mainly, our simulation data show that the arrangement of heterotypic Cx43/Cx45, which is suggested by the expression patterns of cardiac Cxs, would be consistent with the principal role of the AV node–to delay the conduction of electrical signals from the atria to the ventricles. In addition, we demonstrate that the V_j_ gating of heterotypic GJ channels could have a protective effect against re-entrant arrhythmias, to which the AV node is highly susceptible.

### Mechanisms of the drift of spiral waves

The drift of spiral waves in an excitable medium has been considered in multiple theoretical studies. These have shown that this phenomenon can be induced by different mechanisms, such as inhomogeneity^45–47^, anisotropy^48,49^ or by the boundary^50,51^ of the medium. Due to its role in arrhythmic processes (rev. in^52^), many authors have addressed the drift of spiral waves in cardiac tissue in both theoretical^53^ and experimental studies^21^. Studies have demonstrated that the drift can be induced by anatomic inhomogeneity^54^, changes in temperature^55^ or external electric^56,57^, and even optical, stimulation^29,58^. Some studies have also demonstrated a putative role of junctional coupling in the dynamics of spiral waves in cardiac tissue. In^59,60^, the authors showed that bepridil-induced changes in junctional coupling destabilize the re-entrant spiral waves, thus leading to their drift and termination. In this study, we have demonstrated that changes in the g_j_ and the subsequent drift of spiral wave in cardiac tissue could be induced not just by pharmacological agents but by the V_j_ gating of highly V_j_-sensitive heterotypic GJ channels. Because the drift of the rotor was not observed under the control conditions, in which simulated 2D tissue was connected through non-gated GJs, we suppose that gating-induced spatiotemporal inhomogeneities near the tip of the rotor are the most likely mechanistic explanation for the observed drift in our simulation experiments.

### Implications of dynamic coupling for cardiac modelling

A vast majority of cardiac modelling studies assume that the g_j_ does not depend on membrane or trans-cellular voltages. As a result, a conduction tensor in equations for cardiac tissue is usually assumed to be constant at each spatiotemporal point. However, our study shows that in some cases this assumption is not correct and a more detailed representation of cell coupling is necessary. We provide a model to describe dynamic cell coupling that accounts for the membrane excitability of discrete cells and the V_j_ gating of GJ channels. Note, however, that such an approach is computationally challenging, mainly because the evaluation of the dynamic g_j_ using 4SM requires an additional integration of a system of four linear differential equations at each simulated time step. In this study, to avoid any discrepancies associated with insufficient numerical stability, we applied a matrix exponential approach for the integration of 4SM, which is a computationally costly procedure. Thus, accounting for the dynamic gating alone required ~ 20-fold more computation time than using a simple model in which g_j_ is assumed to be a constant. Another reason for the very high computational costs using our model is that the evaluation of GJ channel V_j_ gating using 4SM requires knowing the membrane voltages of the neighbouring cardiomyocytes. Therefore, 4SM is not easily amenable to the bidomain approach, which approximates cardiac tissue as a continuum medium. For this reason, to account for GJ channel gating, we simulated the behaviour of discrete cells, which is less computationally efficient than the bidomain approach. Thus, to further investigate the putative effects of dynamic cell coupling, it will be important to develop new and more efficient numerical approaches. Possible strategies to increase the computation speed might include faster integration schemes, pre-calculation and tabularization of the g_j_ values, homogenization of the 4SM model, or using hybrid methods, such as those proposed in^61^.

## Methods

### Cell culturing and electrophysiological recordings

Experiments were performed in Novikoff cells, endogenously expressing Cx43 and HeLa cells, exogenously expressing Cx45. Cell cultures were grown in DMEM supplemented with 10% foetal calf serum, 100 mg per ml streptomycin and 100 units per ml penicillin. The cells were maintained in a CO_2_ incubator (5% CO_2_) at 37 °C temperature.

Electrophysiological recordings were performed in pairs of cells, one of which was expressing Cx43, and another one Cx45. The cells were grown on coverslips which were bathed in modified Krebs–Ringer solution during the experiment (in mM) 140 NaCl, 4 KCl, 1 MgCl_2_, 2 CaCl_2_, 2 CsCl, 1 BaCl_2_, 5 HEPES, 5 glucose, 2 pyruvate, pH 7.4. The pipettes were filled with a modified Krebs–Ringer solution, which contained (in mM) 130 CsCl, 10 NaAsp, 0.26 CaCl_2_, 1 MgCl_2_, 2 BAPTA, 5 HEPES, pH 7.3. The junctional currents were measured using a dual whole-cell patch clamp method. The signals were acquired and analyzed using path clamp amplifiers (HEKA, Holliston, MA), data acquisition hardware (National Instruments, Austin, TX) and custom-made software^62^.

### The mathematical/computational model of propagation of excitation in cardiac tissue connected through dynamically regulated GJ channels

**Figure 6:**
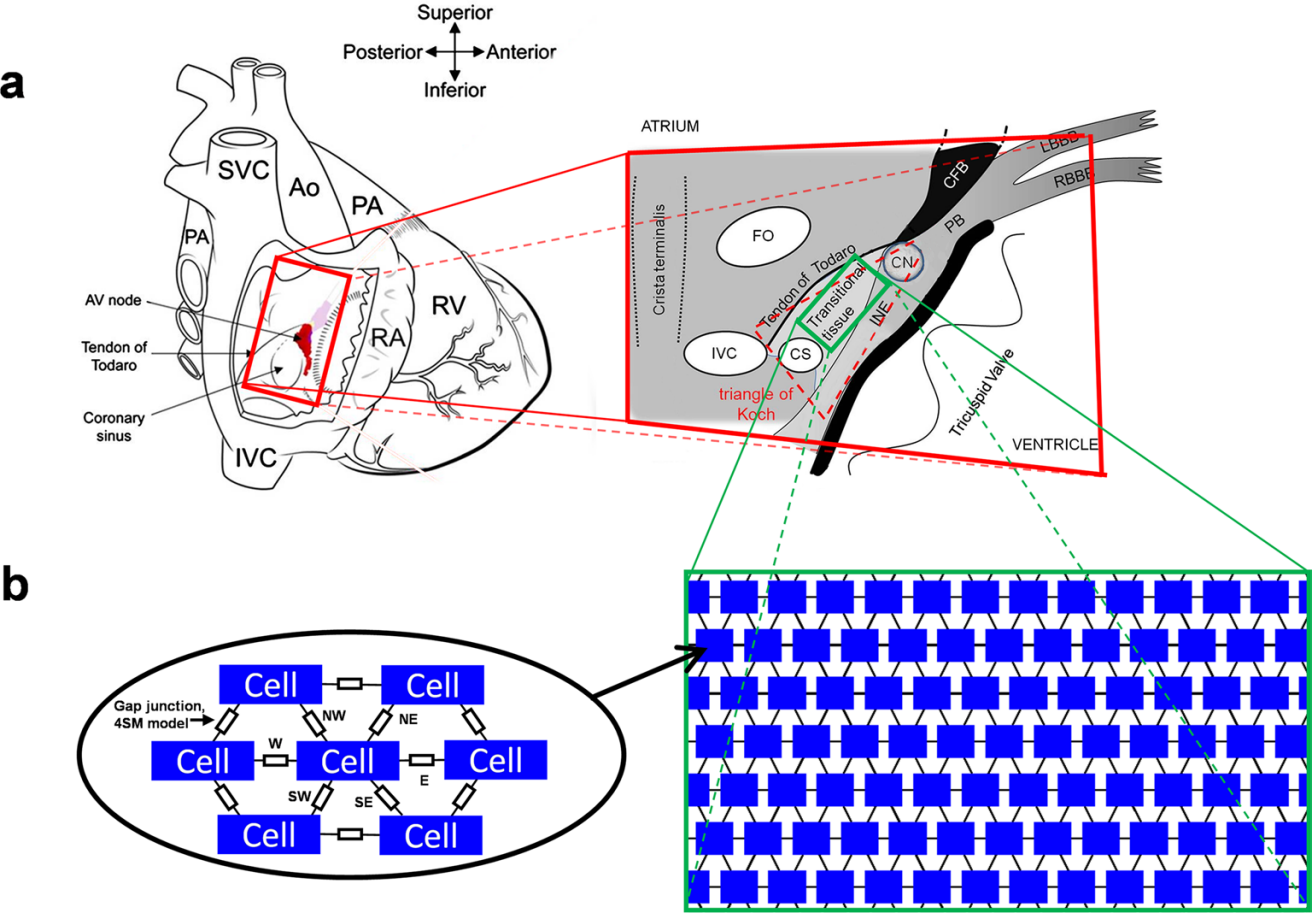
The schematics of the model of cardiac tissue. (**a**) The simulated region is the transitional tissue zone (the green rectangle in the panel on the right side) of the atrioventricular node (AV) that expresses both Cx43 and Cx45. Modified from Fig. 1a in^6^ (covered by the Creative Commons licence). (**b**) A brick-wall-like hexagonal connection structure of tissue of discrete cardiomyocytes. The letters near the junctions (left side) denote their spatial orientation regarding the central cell (NW (west), NE (northeast), E (east), SE (southeast), SW (southwest), and W (west)). The boundary conditions of the simulated 2D tissue (right side) are assumed to be periodic. The dimensions are not to scale.

To investigate the dynamic changes of the junctional conductance (g_j_) and their influence on the propagation of excitation in the myocardium, we developed a mathematical/computational model which combined the membrane excitation of cardiomyocytes and the V_j_ gating of the GJ channels. We were modelling the transitional tissue zone of the AV node (Fig. 6a). In our model of the 2D tissue, the cardiomyocytes were arranged in a brick-wall-like structure with a hexagonal connectivity pattern (Fig. 6b). The boundary conditions were assumed to be periodic.

To describe the excitability of the cardiomyocytes, we used the Fenton–Karma model^17^. The Fenton–Karma model can adequately reproduce cardiac restitution properties, which are important in modelling the dynamics of spiral waves and fibrillation-like processes. In addition, there is a good balance between accuracy and computational efficiency and, therefore, it is suitable for modelling large clusters of cardiomyocytes. We combined these equations with our previously published 4SM model^14^, which can adequately describe the kinetics of g_j_ in response to V_j_ transients. The changes in the membrane voltage of the cardiomyocytes (V_m_) were described by the following set of equations:

$$\frac{{dV}_{m}}{dt}=\frac{{I}_{all}}{{C}_{m}};$$

$${\mathrm{I}}_{\mathrm{all}}={\mathrm{I}}_{\mathrm{ion}}+{I}_{j}+{I}_{ext};$$

$${\mathrm{I}}_{\mathrm{j}}={\sum }_{d\in D}{\mathrm{I}}_{{\mathrm{j}}_{\mathrm{d}}};$$

$${\mathrm{I}}_{{\mathrm{j}}_{\mathrm{d}}}=\left({V}_{{m}_{d}}-{V}_{m}\right)\cdot {g}_{j};$$

$$D=\left\{W,NW,NE,E,SE,SW\right\}.$$

Here, C_m_ is the membrane capacitance and I_all_ is the sum of all the currents in a cell, which consists of all the ionic currents of the cell membrane, the I_ion_, junctional current I_j_ and the external stimulation current I_ext_. The junctional conductance, g_j_, depends on the transjunctional voltage gradient $\left({V}_{{m}_{d}}-{V}_{m}\right)$, and is evaluated using the 4SM; V_m_, being the membrane potential of the cell, ${V}_{{m}_{d}}$, being the membrane potential of the adjacent cell, oriented in the direction d; with the letters in set *D* corresponding to spatial orientation according to the reference cell: W (west), NW (northwest), NE (northeast), E(east), SE (southeast), SW (southwest) (Fig. 6b, left).

A more comprehensive description of the model and its parameters is presented in the Supplementary Information.

The numerical solution of the presented equations was performed using the Euler method with a 0.02 ms time step. To accelerate the simulations, we developed a performance-optimized model by exploiting the GPU, the coalesced memory access and the out-of-core pattern for big data using the Zarr format with BLOSC and LZ compression schemes^63^.

### Supplementary Information

Supplementary Information.

Supplementary Legend.

Supplementary Legend.

Supplementary Video 1.

Supplementary Video 2.

## Data Availability

The datasets used and/or analysed during the current study are available from the corresponding author on reasonable request.

## Code availability

The codes for the implementation of the presented mathematical/computational models are presented in the GitHub repository: https://github.com/KestutisMa/CardioSim/.
