# Supplementary material for: The role of the Cx43/Cx45 gap junction voltage gating on wave propagation and arrhythmogenic activity in cardiac tissue
Source: Sci Rep. 2023 Sep 8;13:14863. doi: 10.1038/s41598-023-41796-w (PMC10491658; doi:10.1038/s41598-023-41796-w)
Supplement: Supplementary file 1 — Supplementary Information. [file 41598_2023_41796_MOESM1_ESM.pdf]

The role of the Cx43/Cx45 gap junction voltage gating on wave propagation and arrhythmogenic activity in cardiac tissue

Kestutis Maciunas[1], *Mindaugas Snipas*[1,2,*], *Tadas Kraujalis*[1,3], *Lina Kraujalienė*[1], *Alexander V. Panfilov*[4,5]

[1] Institute of Cardiology, Lithuanian University of Health Sciences, Lithuania
[2] Department of Mathematical Modelling, Kaunas University of Technology, Lithuania
[3] Department of Applied Informatics, Kaunas University of Technology, Lithuania
[4] Department of Physics and Astronomy, Ghent University, Belgium
[5] Department of Cardiology, Leiden University Medical Center, Leiden, The Netherlands
[*]Correspondence: email: mindaugas.snipas@ktu.lt

Supplementary methods

Fenton-Karma model

To simulate the membrane excitability of the cardiomyocytes, we used the Fenton-Karma model[1]. The model describes current flow through the membrane, I_{ion}, and three gating variables, u, v and w ($0 \leq u, v, w \leq 1$):

$$I_{\text{ion}} = I_{fi}(V_m; v) + I_{so}(V_m) + I_{si}(V_m; w);$$

$$u = (V_m - V_0)/(V_{fi} - V_0).$$

Here, I_{fi} represents fast inward, I_{so} represents slow outward and I_{si} represents slow inward currents that resemble Na^+, K^+ and Ca^{2+} currents, respectively; V_m is the membrane potential, V_{fi} is the Nernst potential of the I_{fi} current, and V_0 is the resting membrane potential.

The current variables are scaled to the membrane capacitance, C_m, as follows:

$$J_{fi} = I_{fi}/\left(C_m \cdot (V_{fi} - V_0)\right);$$

$$J_{so} = I_{so}/(C_m \cdot (V_{so} - V_0));$$

$$J_{si} = I_{si}/(C_m \cdot (V_{si} - V_0)).$$

Then, the model is described by three Liénard-type differential equations:

$$\partial_t u = \nabla(D\nabla u) - J_{fi}(u; v) - J_{so}(u) - J_{si}(u; w);$$

$$\partial_t v = \Theta(u_c - u) \cdot (1 - v)/\tau_i^-(u) - \Theta(u - u_c) \cdot v/\tau_v^+;$$

$$\partial_t w = \Theta(u_c - u) \cdot (1 - w)/\tau_w^- - \Theta(u - u_c) \cdot w/\tau_w^+.$$

Here, a diffusion tensor D is a diagonal matrix whose diagonal elements are equal to 0.001 cm^2/ms; $\Theta(x)$ is the Heaviside step function, defined as $\Theta(x) = 1$ for $x \geq 0$, and $\Theta(x) = 0$ for $x < 0$.

The scaled current variables are calculated as follows:

$$J_{fi}(u; v) = -\frac{v}{\tau_d} \cdot (1 - u) \cdot (u - u_c) \cdot \Theta(u_c - u_c);$$

$$J_{so}(u) = \frac{u}{\tau_0} \cdot \Theta(u_c - u) + \frac{1}{\tau_r} \cdot \Theta(u - u_c);$$

$$J_{si}(u; w) = -\frac{w}{2\tau_{si}} \cdot \left(1 + \tanh\left[k \cdot \left(u - u_{c,si}\right)\right]\right).$$

To accurately reproduce the CV restitution curve, it is necessary to define the time constant that governs the reactivation of the fast inward current separately:

$$\tau_i^-(u) = \Theta(u - u_v) \cdot \tau_{v1}^- + \Theta(u_v - u) \cdot \tau_{v2}^-.$$

In most of our simulations, we used the following set of model parameters (denoted as Set 3 in Table 1 of [2]): τ_{v+} = 3.33 ms, τ_{v1-} = 19.6 ms, τ_{v2-} = 1250 ms, τ_{w+} = 870 ms, τ_{w-} = 41 ms, τ_d = 0.25 ms, τ_0 = 12.5 ms, τ_{si} = 30 ms, k = 10, C_m = 1 μF/cm^2, $u_{c,si}$ = 0.85, u_c = 13, u_v = 0.04, V_{fi} = 15 mV, V_0 = -85 mV.

To avoid the breakup of the wave-front of excitation in our spiral wave experiments, these parameters were modified based on[3]: τ_{v+} = 3.33 ms, τ_{v1-} = 9 ms, τ_{v2-} = 8 ms, τ_{w+} = 250 ms, τ_{w-} = 60 ms, τ_d = 0.395 ms, τ_0 = 9 ms, τ_{si} = 29 ms, k = 15, C_m = 1 μF/cm^2, $u_{c,si}$ = 0.5, u_c = 0.13, u_v = 0.04, V_{fi} = 15 mV, V_0 = -85 mV.

The four-state model of GJ gating

Junctional conductance of Cx43/Cx45 GJ channels was V_j-dependent and was modelled using our previously published four-state model of GJ channel V_j gating. For a more detailed description of the model, we refer to[4]. Examples of model implementation are presented in the supplemental material of that same article. The obtained fits of the V_j gating parameters of the Cx43/Cx45 channels were as follows: $\lambda_1 = 0.0257\ s^{-1}$, $A_{\alpha,1} = 0.0218\ mV^{-1}$, $A_{\beta,2} = 0.0899\ mV^{-1}$, $V_{0,1} = -37.50\ mV$, $\Pi_1 = -1$, $\lambda_2 = 0.0525\ s^{-1}$, $A_{\alpha,2} = 0.0390\ mV^{-1}$, $A_{\beta,2} = 0.0993\ mV^{-1}$, $V_{0,2} = -5.22\ mV$, $\Pi_2 = -1$. The values of the hemichannel conductances were calibrated so that the steady state conductance of the whole channel at $V_j = 0$ mV would be equal to 30 nS: $g_{open,1} = 79.88$ nS, $g_{closed,1} = 6.30$ nS, $g_{open,2} = 73.44$ nS, $g_{closed,2} = 1.13$ nS.

The model of VCT monolayers

To generate realistic patterns of high and low-conductance zones in cardiac tissue, we used a model of VCT monolayers[5]. This methodology is based on the Glazier-Graner-Hogeweg model, which is based on the assumption that cardiac cells maintain their volume and interact with each other via specific adhesion forces. The idea behind the model is that cells are allowed to change their shape to minimize the total energy of the system. Cells are arranged in a regular lattice pattern, and cellular motility is evaluated using an iterative Markov chain Monte Carlo algorithm. For a more detailed description of the model and its parameters, we refer to[5].

References

1 Fenton, F. & Karma, A. Vortex dynamics in three-dimensional continuous myocardium with fiber rotation: Filament instability and fibrillation. *Chaos* **8**, 20-47, doi:10.1063/1.166311 (1998).

2 Fenton, F. H., Cherry, E. M., Hastings, H. M. & Evans, S. J. Multiple mechanisms of spiral wave breakup in a model of cardiac electrical activity. *Chaos* **12**, 852-892, doi:10.1063/1.1504242 (2002).

3 Ashikaga, H. & Asgari-Targhi, A. Locating Order-Disorder Phase Transition in a Cardiac System. *Scientific reports* **8**, 1967, doi:10.1038/s41598-018-20109-6 (2018).

4 Snipas, M. *et al.* Four-State Model for Simulating Kinetic and Steady-State Voltage-Dependent Gating of Gap Junctions. *Biophysical journal* **119**, 1640-1655, doi:10.1016/j.bpj.2020.08.032 (2020).

5 Kudryashova, N., Tsvelaya, V., Agladze, K. & Panfilov, A. Virtual cardiac monolayers for electrical wave propagation. *Scientific reports* **7**, 7887, doi:10.1038/s41598-017-07653-3 (2017).
