# Supplementary material for: The role of the Cx43/Cx45 gap junction voltage gating on wave propagation and arrhythmogenic activity in cardiac tissue
Source: Sci Rep. 2023 Sep 8;13:14863. doi: 10.1038/s41598-023-41796-w (PMC10491658; doi:10.1038/s41598-023-41796-w)
Supplement: Supplementary file 2 — Supplementary Legend. [file 41598_2023_41796_MOESM2_ESM.docx]

**V_j_ gating of heterotypic Cx43/Cx45 GJs can induce a drift of the spiral wave rotor in a 2D cardiac tissue.**

Upper panel shows a drift of the spiral wave rotor in a cluster of cells, which contained V_j_-sensitive heterotypic Cx43/Cx45 GJs. In contrast, no drift was observed when all GJs exhibited constant conductances (lower panel).
