# Supplementary material for: The role of the Cx43/Cx45 gap junction voltage gating on wave propagation and arrhythmogenic activity in cardiac tissue
Source: Sci Rep. 2023 Sep 8;13:14863. doi: 10.1038/s41598-023-41796-w (PMC10491658; doi:10.1038/s41598-023-41796-w)
Supplement: Supplementary file 3 — Supplementary Legend. [file 41598_2023_41796_MOESM3_ESM.docx]

**V_j_ gating of heterotypic Cx43/Cx45 GJs can disrupt fibrillation-like processes in a 2D cardiac tissue.**

Simulation in the upper left panel shows disruption of fibrillation-like activity in a cluster of cells, which contained V_j_-sensitive heterotypic Cx43/Cx45 GJs. V_j_ gating-induced changes of g_j_ can be observed in the upper right panel. Control experiment (lower panels) in a cluster that contained only non-gated channels did not show the disruption of fibrillation-like activity.
